# A dentigerous cyst associated with bilaterally impacted mandibular canines in a girl: a case report

**DOI:** 10.1186/1752-1947-5-230

**Published:** 2011-06-23

**Authors:** Shawneen M Gonzalez, Peter M Spalding, Jeffrey B Payne, Peter J Giannini

**Affiliations:** 1 Department of Oral Biology, Section of Oral and Maxillofacial Pathology and Radiology, University of Nebraska Medical Center College of Dentistry, 40th and Holdrege Streets, Box 830740, Lincoln, NE 68583-0740, USA; 2 Department of Growth and Development, University of Nebraska Medical Center College of Dentistry, 40th and Holdrege Streets, Box 830740, Lincoln, NE 68583-0740, USA; 3 Department of Surgical Specialties, University of Nebraska Medical Center College of Dentistry, 40th and Holdrege Streets, Box 830740, Lincoln, NE 68583-0740, USA; 4 Department of Oral Biology, Section of Oral and Maxillofacial Pathology and Radiology, University of Nebraska Medical Center College of Dentistry, 40th and Holdrege Streets, Box 830740, Lincoln, NE 68583-0740, USA

## Abstract

**Introduction:**

A dentigerous cyst is the most common developmental odontogenic cyst and is frequently noted as an incidental finding on radiographs. The most common teeth affected are impacted mandibular third molars and permanent maxillary canines. This case involves a dentigerous cyst encompassing the right and left impacted mandibular canines and crossing the midline. This is, to the best of our knowledge, the first reported case of a dentigerous cyst encompassing non-adjacent teeth and crossing the midline.

**Case presentation:**

The patient presented to our orthodontic clinic for treatment of malocclusion. The patient was a 10-year, one-month-old Caucasian girl with a dentigerous cyst encompassing the right and left impacted mandibular canines and crossing the midline.

**Conclusion:**

This case involves an unusual clinical and radiographic presentation of a dentigerous cyst. It shows a new variant of presentation that medical professionals, specifically dentists and radiologists, should be aware of, since a dentigerous cyst crossing the midline has not been previously reported as far as we are aware. This additional knowledge is important for inclusion on differential diagnosis lists and aids in the development of a proper treatment plan.

## Introduction

Dentigerous cysts are the most common developmental odontogenic cysts, accounting for approximately 25% of all odontogenic cysts of the jaws. They are frequently noted as an incidental finding on radiographs because a majority of these cysts are asymptomatic and are most commonly associated with impacted mandibular third molars and permanent maxillary canines [1,2]. A dentigerous cyst presents as a well-defined radiolucent entity surrounding the crown of an impacted tooth. The border of the cyst is continuous with the cemento-enamel junction of the impacted tooth. This radiographic finding is pathognomonic for a dentigerous cyst [2]. The occurrence of dentigerous cysts encompassing multiple teeth is uncommon [3-5]. All of the reported cases to date have involved cysts localized to one quadrant of the jaws and have encompassed adjacent teeth. As a dentigerous cyst enlarges, it displaces the affected tooth or teeth apically. Dentigerous cysts enlarge as a result of the accumulation of fluid between the crown of an unerupted tooth and the reduced enamel epithelium.

## Case presentation

**Figure 1:**
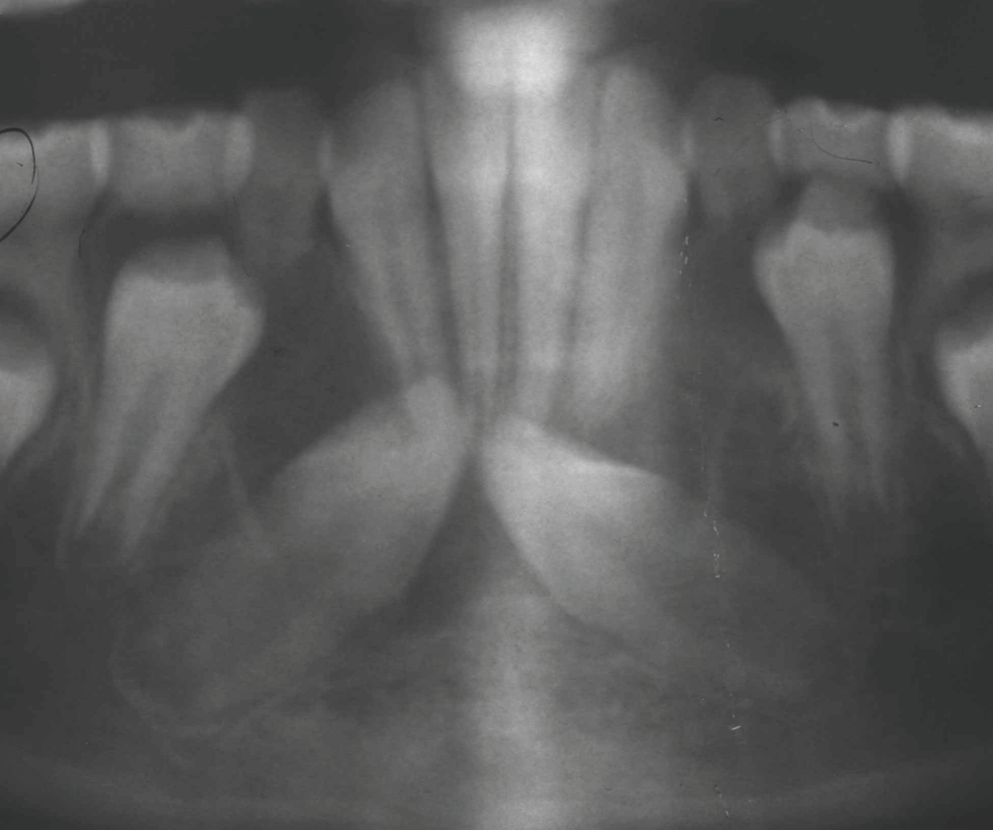
**Cropped pantomograph obtained in July 2008 showing the impacted mandibular canines and a well-defined, radiolucent area around the crown of the impacted mandibular right canine**.

A Caucasian 10-year, one-month-old girl presented to the College of Dentistry for an initial orthodontic work-up. A digital pantomograph and a standardized lateral cephalometric skull radiograph (Planmeca ProMax; Planmeca Oy, Helsinki, Finland) were made. The pantomograph showed impacted mandibular canines, both of which were inclined mesially in a nearly horizontal position and located in the region apical to the mandibular incisors. There was a well-defined radiolucent area evident around the crown of the right mandibular canine that was continuous with the cemento-enamel junction. The radiolucent area measured approximately 20mm in width and 11mm in height. The appearance was consistent with a dentigerous cyst encompassing the crown of the right mandibular canine (Figure 1). The follicle was not discernible around the crown of the left mandibular canine.

**Figure 2:**
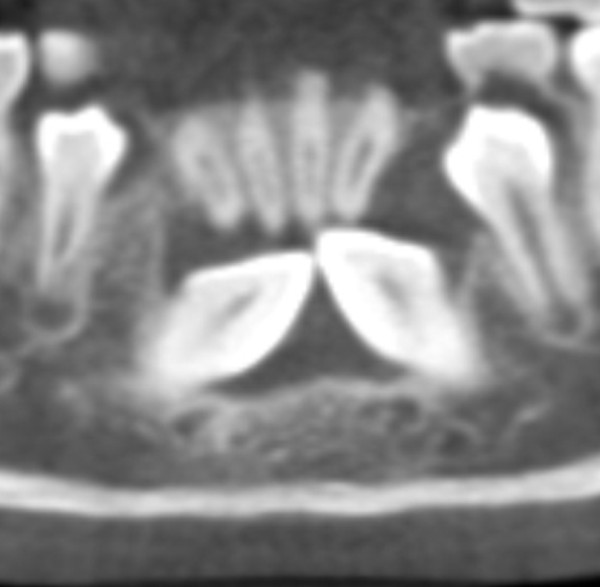
**Reconstructed panoramic slice from cone-beam computed tomography data**. A reconstructed panoramic slice of the anterior mandible shows a well-defined radiolucent area encompassing both impacted mandibular canine crowns with no separation. The border of the radiolucent area appears to be continuous with the cemento-enamel junction of both impacted permanent mandibular canines.

A cone-beam computed tomography scan was ordered and made one month later (iCAT Imaging Sciences, Hatfield, PA, USA) using a 17cm × 16cm × 17cm field of view to evaluate the position of the canines and the dentigerous cyst. The resultant data were reconstructed, and multi-planar and orthoradial views were examined. The coronal and axial slices revealed that the dentigerous cyst was larger than was apparent on the digital pantomograph. The cyst encompassed the crowns of both the right mandibular canine and the left mandibular canine. The border of the dentigerous cyst was continuous with the cemento-enamel junction of the impacted permanent mandibular canines (Figure 2). The appearance was suggestive of a single dentigerous cyst that crossed the midline and encompassed both impacted mandibular canine crowns.

**Figure 3:**
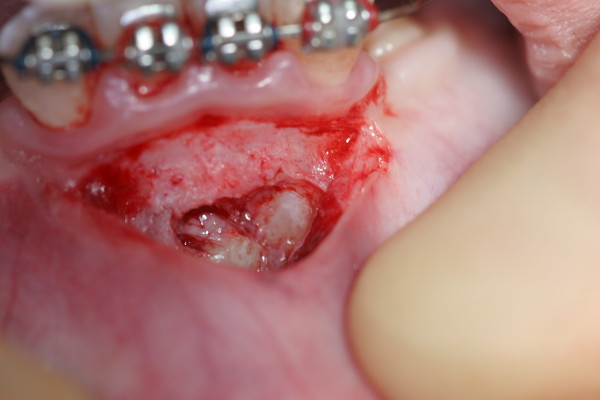
**Clinical photograph from surgical exposure and incisional biopsy**. The photograph shows that the border of the dentigerous cyst is continuous with both of the impacted permanent mandibular canines.

**Figure 4:**
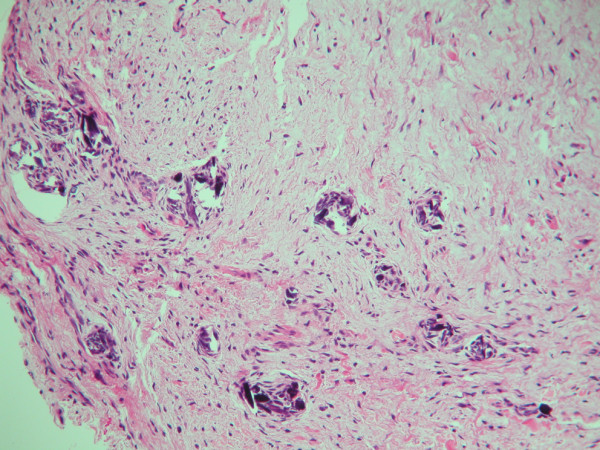
**Hematoxylin and eosin-stained section from incisional biopsy showing non-keratinized, stratified squamous epithelium lining with fibromyxomatous connective tissue consistent with a diagnosis of dentigerous cyst**.

Surgical exposure of the canines with an incisional biopsy of the surrounding dentigerous cyst was performed by a periodontist (JBP) three months after the initial appointment. It was noted that the dentigerous cyst border was continuous with the cemento-enamel junction of both impacted permanent mandibular canines (Figure 3). The incisional biopsy specimen was sent to the University of Nebraska Medical Center College of Dentistry Oral Pathology Biopsy Service for pathologic examination. Hematoxylin and eosin-stained sections of the specimen were prepared. The histopathologic sections showed a cyst lined by thin, non-keratinized, stratified squamous epithelium. The cyst wall consisted of uninflamed fibromyxomatous connective tissue. These findings were consistent with a diagnosis of a dentigerous cyst (Figure 4).

**Figure 5:**
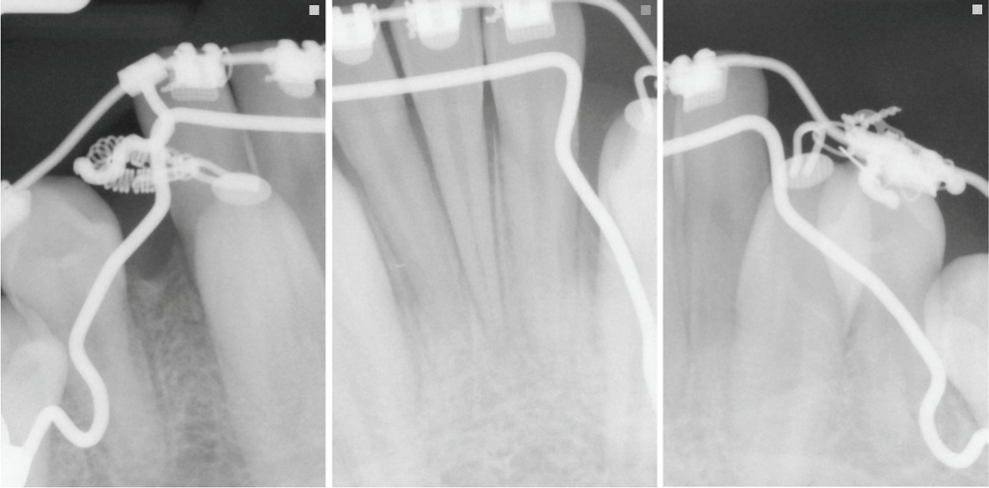
**Peri-apical radiographs obtained in July 2009 showing the bilateral mandibular canines, which depict no recurrence of the dentigerous cyst after partial orthodontic uprighting**.

One year later peri-apical radiographs of the mandibular canines (Figure 5) were made that showed no recurrence of the dentigerous cyst and partial orthodontic uprighting of the canines. At one year after surgery, there was no evidence of recurrence.

## Discussion

There were no previous radiographs showing the patient's mandibular anterior teeth to determine whether the cyst originated as a single dentigerous cyst that enlarged and grew to encompass the other impacted canine. There are other reported cases of a dentigerous cyst enlarging and encompassing other adjacent teeth [3-5]. The other possibility is that both impacted canines had enlarging dentigerous cysts that converged, with consequent resorption of the interposing osseous border to create one large, dentigerous cyst that included both teeth. One previous case report described a patient with two adjacent teeth with enlarging dentigerous cysts that still had a bony separation between the two [6]. This finding would lead to the possibility of one dentigerous cyst that enlarged and encompassed the other impacted canine. However, the radiographic findings in that case report showed the border of the dentigerous cyst to be continuous with the cemento-enamel junction of both impacted permanent mandibular canines. On the basis of this radiographic finding, both of these possibilities cannot be ruled out in the absence of previous radiographs.

## Conclusion

This case describes the presentation of a dentigerous cyst that has not been previously reported. This new presentation shows that a dentigerous cyst can encompass multiple non-adjacent teeth in addition to the possibility that a dentigerous cyst might also cross the midline. Previously, these two variants would lead to a differential diagnosis not including a dentigerous cyst. This is important for medical professionals to be aware of, specifically dentists and radiologists, so that an accurate differential diagnosis can be made to determine the best treatment for the patient.

## Consent

Written informed consent was obtained from the patient for publication of this case report and any accompanying images. A copy of the written consent is available for review by the Editor-in-Chief of this journal.

## Competing interests

The authors declare that they have no competing interests.

## Authors' contributions

SG interpreted the radiographs and the cone-beam computed tomography scan. PS performed the orthodontic movement of the mandibular canines once they were exposed. JP performed the incisional biopsy, curettage, and exposure of the mandibular canines. PG performed the histological examination of the biopsy. All authors read and approved the final manuscript.
